# Serum LPS Associated with Hantavirus and Dengue Disease Severity in Barbados

**DOI:** 10.3390/v11090838

**Published:** 2019-09-09

**Authors:** Kirk Osmond Douglas, Thelma Alafia Samuels, Marquita Gittens-St. Hilaire

**Affiliations:** 1 Faculty of Medical Sciences, University of the West Indies, Cave Hill, BB11000 St. Michael, Barbados; 2 Epidemiology Research Unit, Caribbean Institute for Health Research (CAIHR), The University of the West Indies, Mona Kingston 7, Jamaica; 3 Best-dos Santos Public Health Laboratory, Enmore #6, Lower Collymore Rock, St. Michael BB11155, Barbados

**Keywords:** LPS, lipopolysaccharide, dengue, hantavirus, dengue fever, disease severity, HFRS, HPS, DENV, endotoxin, microbial translocation

## Abstract

Hantavirus and dengue virus (DENV) infections are caused by RNA viruses which infect immune systems’ cells including monocytes, macrophages and dendritic cells and occur year-round in Barbados. A retrospective serological study (2008–2015) was conducted on hantavirus and dengue patient sera confirmed by IgM and IgG ELISA, NS1 and RT-PCR using *Limulus* amoebocyte lysate (LAL) kinetic turbidimetric method to determine serum endotoxin levels. Hantavirus patients were categorized into two groups, namely (a) hospitalized and (b) non-hospitalized. Dengue patients were categorized into 3 groups using 2009 WHO dengue guidelines (a) severe dengue (SD), (b) hospitalized non-severe dengue (non-SD) and (c) non-hospitalized non-SD. Statistical analyses were conducted to determine the association of endotoxin levels with hantavirus disease severity based on hospitalization and dengue disease severity. Serum endotoxin levels are associated with hantavirus disease severity and hospitalization and dengue disease severity (*p* < 0.01). Similar studies have found an association of serum endotoxin levels with dengue disease severity but never with hantavirus infection. Co-detection of hantavirus- and DENV-specific IgM in some patients were observed with elevated serum endotoxin levels. In addition, previous studies observed hantavirus replication in the gut of patients, gastrointestinal tract as a possible entry route of infection and evidence of microbial translocation and its impact on hantavirus disease severity. A significant correlation of serum endotoxin and hantavirus disease severity and hospitalization in hantavirus infected patients is reported for the first time ever. In addition, serum endotoxin levels correlated with dengue disease severity. This study adds further support to the role of endotoxin in both hantavirus and dengue virus infection and disease severity and its role as a possible therapeutic target for viral haemorrhagic fevers (VHFs).

## 1. Introduction

Dengue viruses (DENVs) and hantaviruses are global health threats accounting for 58.4 million annual dengue cases and as many as 0.2 million annual hantavirus infections respectively [1,2]. With both dengue and hantavirus infection, the role of the vector is important namely the mosquito primarily *Aedes aegypti* and rodents (Muridinae family e.g., rats and mice) respectively. Transmission of DENV and hantaviruses is supported by anthropogenic activities that increase population growth, urbanization, air travel and climate change.

Hantaviruses are single stranded (SS) negative-sense RNA viruses approximately 120–160 nm in diameter from the Hantaviridae virus family [3,4]. Hantaviruses can be separated into two groups, Old World (Seoul (SEOV), Dobrava (DOBV), Puumala (PUUV) and Hantaan (HTNV)) and New World (Prospect Hill (PHV), Andes (ANDV) Sin Nombre (SNV), etc) based on the M segment (nucleotides 1987–2315) [3,4]. DENV and hantaviruses are both RNA viruses, which infect similar human host cells. DENV infection can lead to dengue fever (DF), dengue haemorrhagic fever (DHF) and dengue shock syndrome (DSS). Whilst hantavirus infection can lead to three main clinical diseases namely nephropathica epidemica (NE), haemorrhagic fever with renal syndrome (HFRS) and hantavirus pulmonary syndrome (HPS) [5]. DENV and hantavirus infections have similar clinical symptoms making the differential diagnosis difficult without the use of clinical laboratory diagnostic testing. These symptoms can include fever, myalgia, arthralgia, nausea, vomiting, rash, headache, bleeding manifestations, abdominal pain, and jaundice. Their clinical symptoms are so similar that initially researchers thought DHF epidemics in SE Asia were due to epidemic haemorrhagic fever caused by hantaviruses [6,7,8].

Dengue has been endemic in Barbados for over 30 years with the circulation of all four serotypes of DENV [9,10,11,12,13,14,15,16,17]. DENV are transmitted year-round and dengue epidemics have occurred in 1995, 1997, 2001, and 2007 [11,12,14]. Notably DHF in Barbados does not appear to be as prevalent as in Asian countries where dengue is more severe [18]. Though dengue has been endemic in the Caribbean for several decades some of the host factors influencing dengue disease severity and hantavirus disease severity in the region remain unknown. DENV infection can impact on the clinical haematological profile resulting in leukopenia, thrombocytopenia, and haemorrhagic manifestations profile has been well established [19,20,21,22]. The basis for assessing dengue disease severity using WHO 2009 guidelines is founded on research conducted on host responses during severe and non-severe dengue [23,24].

Hantavirus transmission in Barbados has been detected since 2001 among febrile persons under investigation for acute DENV infection and in wild rats [25,26]. Hantavirus infections have remained endemic in Barbados since then however the identity of the circulating strain(s) has proven elusive. Hantavirus infections are detected using Focus SelectDx™ hantavirus ELISA IgM and IgG kits which use a recombinant nucleoprotein (rNP) mixture for detecting antibodies to a broad range of hantavirus strains. including SEOV, HTNV, PUUV, DOBV, and SNV. In the Caribbean serological and molecular evidence of hantavirus transmission exists for Grenada, Trinidad and Cuba (Sin Nombre strain positive by RT-PCR) however no extensive epidemiological data on hantavirus infection exists for the Caribbean [27,28,29,30]. Recent hantavirus outbreaks in adjacent regions including 4 fatal HPS cases observed in French Guiana (Maripa virus) in 2016 enhance the risk of new and more lethal hantavirus strains entering the Caribbean region via trade and travel [31].

Lipopolysaccharide (LPS) or endotoxin is the immunologically relevant component of Gram-negative bacteria and elicits an immune response if present within the body [32]. Gram-negative bacteria shed endotoxin during their normal growth [33]. It is comprised of two segments namely a lipid portion (lipid A) and polysaccharide portion (O specific chain and core). Lipid A segment is the most highly conserved whilst the O specific chain is the most variable [34]. The presence of bacterial endotoxin in serum or blood can arise through (a) active acute/systemic bacterial infection within the body or (b) its translocation (microbial translocation—MT) from the human gut colonized by large numbers of Gram-negative bacteria [34,35]. Human host generated response in the clearance of LPS involves the production and binding of serum proteins including lipopolysaccharide binding protein (LBP), bacterial/permeability increasing protein and sCD14, host defence peptides, and chylomicrons [34,36,37,38,39]. LBP and sCD14 can enhance the pathogenicity of endotoxin as they induce the production of inflammatory cytokines by monocytes [34]. The cytokine production in the presence of endotoxin is key in the pathogenesis of the clinical syndromes of shock and multiple organ failure [40]. Several cytokines are released in response to the presence of endotoxin including tumour necrosis factor (TNF), interleukin (IL)-1, IL-6, IL-8, IL-10 [34,40].

DENV and hantavirus infections in humans target monocytes and tissue macrophages which are in highest concentration in the gut-associated lymphoid tissue (GALT). DENV and hantavirus replication in these monocytes and macrophages can lead to the movement of soluble factors across the intestinal lining resulting in increased endotoxin/LPS levels in the blood and increased severity of disease [41,42]. LPS can inhibit DENV infection of macrophages/monocytes but only if present prior to DENV adsorption [43]. LPS induction of flaviviral disease severity has been previously observed in West Nile virus (WNV) infections [44]. Bacterial endotoxin contributes to the penetration of WNV from the blood into the central nervous system (CNS) causing a mild WNV infection to become a severe lethal encephalitis [44]. Human immunodeficiency virus (HIV) also targets monocytes and high endotoxin levels are associated with HIV replication in the GALT due to MT [45,46]. DENV replication in intestinal macrophages may lead to a pro-inflammatory environment in which the epithelial cells are disrupted allowing the passage of soluble factors from the gut into the blood [47]. Some hantavirus infections can have clinical presentations like acute appendicitis suggesting replication in the human intestine [48,49,50]. The detection of hantavirus antigen in the gut epithelium [49,50] raised the possibility of MT occurring during acute hantavirus infection but a correlation of MT and disease severity was later confirmed in Argentine HPS patients [42]. To date no studies have been reported on the role of serum endotoxin in hantavirus or dengue infection and no host markers and their association with disease severity has been investigated among hantavirus patients in the Caribbean.

### Objectives

We aimed to investigate the role of serum endotoxin levels (a) in hantavirus disease severity, (b) in dengue disease severity in the Caribbean using 2009 WHO dengue guidelines and (c) and their correlation with clinical parameters among severe dengue (SD) and hospitalised non-severe dengue (non-SD) patients.

## 2. Methods

### 2.1. Hantavirus Serum Endotoxin Study: Cases and Controls Selection

The relevant ethical approval was obtained from the University of the West Indies (UWI)/Ministry of Health of Barbados Internal review Board (IRB) on 11 July 2013 and the Ethics Committee at the Queen Elizabeth Hospital (QEH), Martindale’s Road, St. Michael, Barbados on 19 August 2013 prior to the start of data collection and granting access to database and the use of archived frozen sera samples. No informed patient consent was necessary for this study as it was retrospective in nature and only frozen, archived, acute hantavirus and DENV infected patient sera were used.

**Figure 1:**
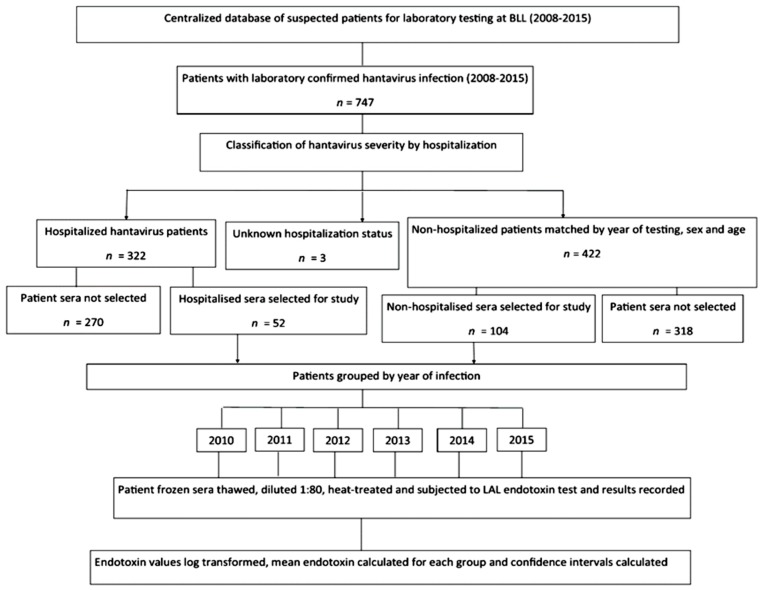
Flow chart of the investigation of serum endotoxin and hantavirus disease severity.

Using centralized database at Best-Dos Santos Public Health Laboratory, St. Michael, Barbados, a list of all patients (*n* = 756) with confirmed hantavirus infection by IgM serology and year (2008–2015) were selected (Figure 1). Hantavirus cases were confirmed by detection of hantavirus-specific IgM and IgG in patients’ serum (IgM in samples within 5–15 days of illness) with Focus DxSelect™ hantavirus IgM and IgG ELISA kits (Focus Diagnostics, Cypress, CA, USA) following the manufacturer’s instructions. The Focus Diagnostics Hantavirus DxSelect™ kit uses a cocktail of baculovirus-derived recombinant nucleoprotein (NP) of hantavirus strains. Using a rNP cocktail allows for detecting antibodies to a broad range of hantavirus strains. The Focus Diagnostics Hantavirus DxSelect™ ELISA kit will detect antibodies to the most clinically relevant pathogenic strains of Hantaviruses, i.e., SEOV, HTNV, PUUV, DOBV, and SNV. A clinical laboratory hantavirus case was assigned according to the CDC hantavirus case definition for non-Hantavirus Pulmonary Syndrome (HPS) specifically a) “the detection of hantavirus-specific IgM [51]. Confirmatory hantavirus testing was performed on some hantavirus cases using immunofluorescence assays and pseudotype focus neutralisation test (pFRNT) [52]. All patients included in the endotoxin study were hantavirus IgM ELISA positive confirming acute infection.

For each hospitalised hantavirus patient (Group 1, *n* = 52) two age-, sex- and year- matched non-hospitalised hantavirus patients (Group 2, *n* = 104) were selected from the list to increase the power of the study (Figure 1). The power of the study was calculated as detailed in Table 1. Only hospitalised hantavirus patients and matching non-hospitalised hantavirus patients with frozen sera and of a sufficient volume (>80 µL) were included into the endotoxin study. Ideally matching controls of non-infected patients (age- and sex- and year-matched) would have been included in the analysis however due to the retrospective nature of the study it would have been impractical to obtain these control age- and sex- and year-matched samples from 2008–2015. The clinical symptom frequencies were calculated for hospitalised hantavirus infected patients.

### 2.2. Serum Endotoxin Study: Dengue Patient Selection

**Figure 2:**
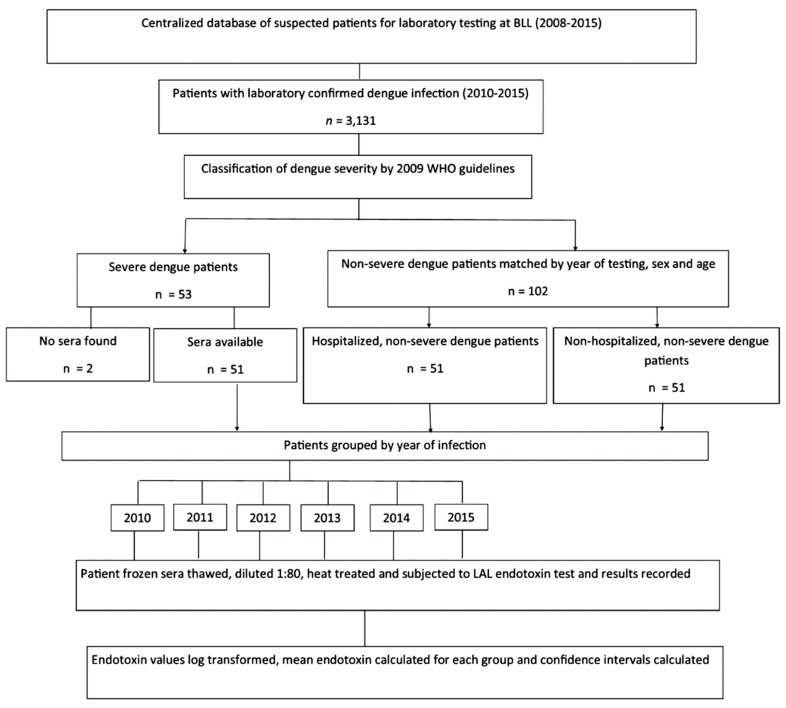
Investigation of serum endotoxin and dengue disease severity method flowchart.

A list of 3131 patients with confirmed DENV infection (2010–2015) was generated from a centralized patient database at Best-Dos Santos Public Health Laboratory and grouped by year of infection (Figure 2).

DENV infection was confirmed by dengue specific IgM and IgG enzyme linked immunoassay (ELISA) (Focus Diagnostics, CA, USA), NS1 antigen kit and or real time reverse transcriptase polymerase chain reaction (rRT-PCR) from the samples obtained at day of admission or in acute and/or convalescent samples. DENV-specific IgG was determined using Focus DxSelect™ DENV IgG ELISA to see if a previous DENV infection occurred. Patients were classified using the 2009-WHO-criteria and the expert physician’s judgment of disease severity. All patients with severe dengue (SD) between 2010 and 2015 were selected (*n* = 53 patients) but two (2) were excluded due to a missing serum sample for the SD patient or the absence of both a suitable hospitalized and non-hospitalized non-severe dengue (non-SD) age-, sex- and year-matched patient serum sample. Patients were categorized into three groups, Groups 1, 2 and 3. Group 1 (*n* = 51) was comprised of all SD patients from 2010 to 2015 with suitable matching non-SD patients. Groups 2 and 3 were age-, sex- and year-matched non-SD patients. Group 2 (*n* = 51) was comprised of hospitalized non-SD patients and Group 3 (*n* = 51) was comprised of non-hospitalized non-SD patients. Archived frozen sera samples from identified DENV infected patients were collected and stored in marked cryogenic container for the research investigation. Only SD patients and age-, sex- and year-matched non-SD patients with frozen sera and of a sufficient volume (>80 µL) were included into the endotoxin study. The sample size of the SD patients was set based on the number of limited SD patients in the study period. A ratio of 2:1 (non-SD patients: SD patient) was determined to increase the power of the study.

### 2.3. Analysis of Clinical Laboratory Parameters and Dengue Disease Severity

The data regarding clinical characteristics including signs and symptoms, biochemical laboratory data, complications in the form of DHF and DSS and outcome in terms of numbers of days admitted and mortality if any were recorded on a specially designed data abstraction form using Epi Info 7 software. This was done to ensure stringency of data entry and data quality. The data used for analysis was taken from the first day of admittance to QEH to reflect the admission protocol against the data collected. This permits the evaluation of endotoxin and grading of patient disease severity with DENV infection. Clinical, haematological and biochemical parameters were evaluated by determining how many age-, sex- and year- matched patients from the SD patient group and hospitalized non-SD patient controls had out of range values. Each parameter for each patient was evaluated for out of range status thus converting the continuous variable to categorical variable. This was done rather than assessing the means as clinically relevancy of the comparison was sought. The proportion of severe dengue patients with out of range clinical parameters were compared with hospitalized dengue group using McNemar’s test due to the small sample sizes involved and paired sample data used [53]. All parameters were tested at the 95% confidence level.

### 2.4. Endotoxin Determination in Dengue—and Hantavirus-Infected Patient Sera

Serum samples were stored at −80 °C and numerous repetitive freeze–thaw cycles avoided. Due to possible interference sera samples were diluted accordingly (1:80) with *Limulus* Amoebocyte Lysate (LAL) reagent water and heat-inactivated at 55–60 °C in a dry-bath incubator for 30 min. Known concentrations of *Escherichia coli* LPS were diluted in endotoxin free water and served as the standard curve (0.001–1 endotoxin units per millilitre (EU/mL)). Each bottle of turbidimetric LAL lysate was reconstituted with 5 mL of GlucoShield^™^ buffer (Associates of Cape Cod Incorporated, East Falmouth, MA, USA) which acts to block any non-specific reaction of LAL reagent and ensure specificity to bacterial LPS. LPS was determined with a commercially available *Limulus* Amoebocyte Lysate (LAL) kinetic assay (Associates of Cape Cod Incorporated, USA) following the manufacturer’s instructions. For the LPS standard aliquots of 100 μL of each LPS dilution (0.001–1 EU/mL) was added in duplicate to depyrogenated tubes. One hundred microliters of each sample of diluted and heat-treated serum was added to a depyrogenated tube. To each of these tubes 100 μL of turbidimetric LAL Reagent was added, vortexed for 3 s and placed into the turbidimetric reader for incubation at 37 °C ± 1 °C for at least 1 h. The standard and sera samples were incubated with the appropriate turbidimetric LAL lysate under the manufacturer’s recommended incubation conditions. For each assay run validity care was taken to ensure the endotoxin standard curve was >0.980, the negative controls contained no detectable endotoxin and the positive sample controls were between 50 and 200%.

### 2.5. Data Treatment and Statistical Analysis

The endotoxin results for each sample was recorded in an Excel spreadsheet. The endotoxin results in EU/mL were converted to picogram per millilitre (pg/mL) and log transformed to standardize the data distribution to a log-normal distribution and permit standard statistical analysis [54,55]. The log transformed mean endotoxin levels for each group were calculated as previously described [54,55]. Using 99% confidence intervals method the statistical difference between the means was evaluated [54,55].

## 3. Results

### 3.1. The Association of Serum Endotoxin Levels and Hantavirus Disease Severity

A significant association of serum endotoxin levels with hantavirus infection and hospitalization was observed (Table 2). The mean endotoxin of hospitalized hantavirus patients (76.02 (48.10–120.16) pg/mL) was significantly higher than non-hospitalized hantavirus patients (24.33 (18.07–32.76) pg/mL) (*p* < 0.01). A higher proportion of hantavirus infected females than males were involved in this study (67.3% vs. 32.7%, χ^2^ = 11.97, *p* < 0.01). A large proportion of hospitalised hantavirus cases (57.7%) experienced gastrointestinal symptoms and vomiting (Table 2). A higher proportion of hospitalised hantavirus patients in the endotoxin study had respiratory symptoms than non-hospitalised hantavirus patients (25.0% vs. 6.7%, χ^2^ = 12.05, *p* < 0.01).

### 3.2. The Association of Serum Endotoxin Levels and Dengue Disease Severity

**Figure 3:**
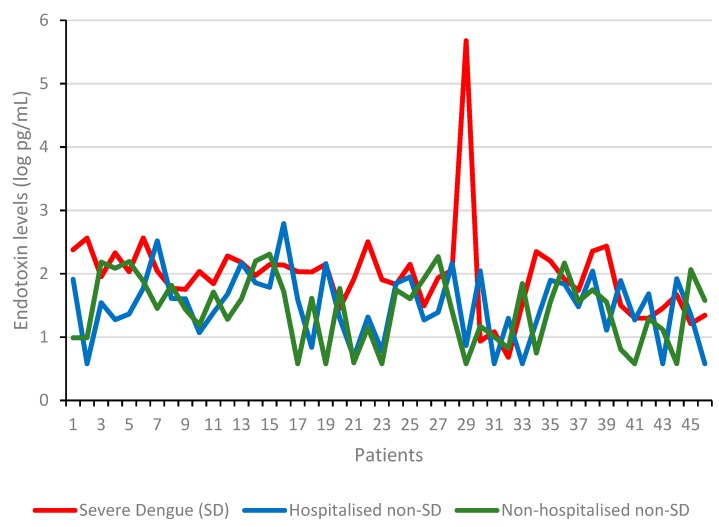
Serum endotoxin levels of SD, hospitalised non-SD and non-hospitalised non-SD patients in Barbados.

A significant association of serum endotoxin levels with DENV infection and disease severity was observed (Table 3). The mean endotoxin of SD patients 86.24 (48.56–153.15) pg/mL was significantly higher than hospitalized non-SD patients 31.55 (20.63 to 48.20) pg/mL, (*p* < 0.01) and non-hospitalized non-SD patients 26.96 (17.60 to 41.30) pg/mL (*p* < 0.01). There was no statistically significant difference between the mean serum endotoxin levels among hospitalized non-severe dengue patients, 31.55 (20.63 to 48.20) pg/mL and non-hospitalized non-severe dengue patients, 26.96 (17.60 to 41.30) pg/mL (*p* > 0.01). A similar proportion of DENV infected females and males were involved in this study (48.1% vs. 51.9%) *p* > 0.01. Serum endotoxin among SD patients trended higher than either hospitalised non-SD and non-hospitalised non-SD patients (Figure 3).

### 3.3. Severe Dengue vs. Hospitalized Non-Severe Dengue

Several haematological and biochemical parameters of severe dengue and hospitalized non-severe dengue patients obtained from the 1st day of clinical presentation to the physician were compared for significant differences. Paired data was absent for several SD cases and matched hospitalized non-SD patients, which reduced the power of the analyses due to missing medical records and or missing clinical data. Both data from a SD case and a sex- and age- matched hospitalized non-SD patient was necessary for analysis. None of the serum clinical chemistry parameters were significantly different between SD cases and matched hospitalized non-SD patients (Table 4). Of the haematological parameters examined with paired data only platelet count and RDWCV (*p* < 0.05, McNemar’s test) were identified as parameters having a significant difference between SD patients and hospitalized non-SD patients (Table 4). Given the large number of hypothesis tests conducted the risk of family wise error (FWE) rate (making false discoveries) was reduced by performing Bonferroni correction and resetting the level of significance to 99.9% or α = 0.001. 

## 4. Discussion

A significant association of mean serum endotoxin and hantavirus infection in addition to hospitalization was observed for the first time ever among hantavirus cases. The mean serum endotoxin levels in hantavirus cases was higher than among non-hospitalized controls. Severe gastrointestinal (GI) symptoms including abdominal pain, nausea and vomiting were previously found in a high percentage of patients with PUUV infection [56,57,58]. This was also observed in this study among hospitalised hantavirus cases suggesting the likelihood of MT in hantavirus infected patients and disease severity. With regards to hantavirus infection the detection of hantavirus antigen using immunohistochemistry (IHC) staining in the biopsied intestines (lamina propria) from Puumala (PUUV) infected patients and similar hantavirus tropism for monocytes and dendritic cells like DENV are suggestive of hantavirus replication in the GALT and microbial translocation [49,50]. It is not known whether hantaviruses set the stage for a secondary bacterial infection or are the cause of inflammation [49,50]. One study investigating the role of serum biomarkers in hantavirus infection and disease severity showed intestinal fatty acid binding protein (I-FABP) and interleukin 6 (IL-6) cytokine levels were associated with disease severity and poor outcome among Argentine HPS patients [42]. Recent evidence of hantavirus infection via the intestinal route has been advanced defying conventional thought of hantavirus infection via infectious aerosols opening a new frontier of hantavirus research [59]. Further studies on the role of MT in hantavirus infection, its role in disease severity in HFRS and HPS is thus warranted.

In this endotoxin study no statistically significant difference in the frequency of gastrointestinal related symptoms was observed between hospitalised and non-hospitalised patients indicating the similarity of clinical presentation regardless of hospitalisation. Though if vomiting is analysed separately there is a statistically significant difference between hospitalised and non-hospitalised patients [52]. This does however underscore the importance of serum endotoxin levels to distinguish between hospitalised and non-hospitalised hantavirus patients adding support for the role of endotoxin in viral infections and disease severity via MT.

For hospitalised hantavirus patients gastrointestinal related, respiratory symptoms and thrombocytopenia were all statistically significant clinical symptoms observed when compared with non-hospitalised hantavirus patients. Respiratory symptoms can be observed in a significant number of HFRS patients mimicking HCPS thus observing a higher frequency of respiratory symptoms among hospitalised hantavirus patients is not unusual [60]. Platelets play a significant role in VHFs and their disease severity and for hantaviruses the intensity of platelet β3 integrin factor is higher patients with more severe HFRS disease [61]. The statistically significant association of thrombocytopenia among with hospitalised hantavirus patients is thus not surprising. A statistically significantly higher proportion of females was observed among hantavirus patients selected in this study. This is also reflected in the overall population infected by hantavirus studies in Barbados during 2008–2016 [52]. The ratio of males to females in the general population of Barbados is effectively 1:1 however the ratio of hantavirus infected females to males is almost 2:1. Qualitative research among hantavirus patients is needed to determine the cause(s) for this disparity between sexes. Possible theories could include the higher percentage of females working in the sugar cane harvesting industry, higher exposure occupational risk working in sugarcane fields and the higher hantavirus seroprevalence among rodents in sugarcane fields and urban areas.

The correlation of serum endotoxin levels with dengue severity observed in our study appears to be consistent with the 2009 WHO dengue guidelines for dengue severity. A correlation of dengue disease severity and LPS levels exist where DENV replication in intestinal macrophages leads to disruption of intestinal integrity and passage of endotoxin from the gut to the blood [47]. The presence of LPS in the blood can permit LPS mediated enhancement of DENV replication and synergistic IFN-α production with consequent modulation of disease progression [41]. A similar effect of LPS and cytokines on dengue disease severity was observed among patients in Brazil with the occurrence of MT and activation of monocytes [62].

Other studies have provided evidence for DENV infection, localized intestinal effects of DENV infection and disease severity. Gastrointestinal bleeding has been identified as a positive associated factor for DSS lending further support to the association of serum endotoxin and dengue disease severity via MT and disruption of the gut epithelium [21,47]. Gastrointestinal bleeding was associated with DSS rather than other types of bleeding including mucosal and cutaneous and is likely expected if microbial translocation and GI epithelial damage occurs with dengue infection [21]. In addition, some studies found abdominal pain or tenderness to be a predictor of severe dengue [63,64]. This clinical sign may be indicative of the site of viral replication in the GALT. An analysis of 306 fatal cases of dengue to highlight clinical presentations as warning signs of fatal DHF showed massive gastrointestinal bleeding was a major observation [65]. Studies using intestinal fatty acid binding protein (I-FABP) as a biomarker of intestinal injury showed a correlation of dengue disease severity and elevated I-FABP levels providing further evidence of DENV replication and MT [66]. Elevated endotoxin levels are present in severe dengue cases compared with non-severe dengue and uninfected controls [67].

DENV infection can impact on the clinical haematological profile resulting in leukopenia, thrombocytopenia, and haemorrhagic manifestations profile has been well established [19,20,21,22]. The parameters identified in our study with significant differences between severe dengue and hospitalized dengue patients were platelet count, PTT and PT. However, these differences were prior to the Bonferroni correction, which reduced the significance due to the very small sample sizes. The parameters analysed were from the 1st day of clinical presentation to the physician and have previously been useful in identifying severe dengue cases. Data from a prospective observational study of Vietnamese children (5–15 years; *n* = 2,301) show that daily platelet counts permit accurate discrimination of patients who develop DSS [68]. A rapid drop in platelet count or thrombocytopenia is a warning sign for dengue severity in 2009 WHO dengue guidelines [69]. Our findings agree with previous studies on coagulation factors as risk factors for DSS development [21,69,70]. Some conflicting results have been obtained. One Thai study examining clinical laboratory findings collected within 72 h of fever onset from a prospective cohort of children found platelet count along with WBCs, haematocrit and percent monocytes were 97% sensitive to predict DSS patients [71]. More studies support platelet counts as predictors of dengue severity [72]. Conversely another study in Thailand found no significant difference in admission haematology laboratory data between both young DHF patients with and without shock concluding that admission haematology data is unable to predict shock [73]. Our results differ from the latter study and may be due to age, study design, different population genetics and viral genetics of DENVs circulating in Asia.

Normal platelet counts range from 150,000 to 450,000 platelets/µL or 150–450 platelets × 10^9^  L^−1^. The action of DENV on platelets and their production have been investigated and reviewed [62,74,75]. During acute infection DENV reduces platelets either by decreasing platelet production or increased platelet destruction [62,74]. DENV can decrease platelet production by inhibition of bone marrow progenitor cell growth [62,75]. DENV also reduces platelet count through direct destruction of platelets by (1) directed binding of anti-dengue NS1 antibodies to platelets leading to complement-mediated lysis [76,77], (2) high levels E-selection on endothelial cells promoting platelet adhesion and clearance [78], (3) direct binding of DENV to platelets via lectin receptors such as CLEC-2 and DC-SIGN [74] and disseminated intravascular coagulation (DIC) [79] and increased apoptosis due to binding of DC-SIGN and caspases [80].

Red blood cell distribution width-coefficient of variation (RDW-CV) is a measure of the variation of the red blood cell volume. This parameter is capable of change due to the storage conditions of the blood as it has been identified as showing statistically significant changes [81]. Changes in RDW-CV can lead to anaemia (lowering of RBCs) where DENV infection leads to haemolysis and bleeding reducing the volume of RBCs [82,83]. With SD/DHF patients, the risk of bleeding would be higher than those that are non-severe dengue patients.

Some studies have recommended the use of platelet transfusions where low platelet levels are observed i.e., 10–20 × 10^9^  L^−1^ without haemorrhage or 50 × 10^9^  L^−1^ with bleeding or haemorrhage. However, evidence exists that show platelet transfusions are not as effective as expected but can lead to several complications including no significant difference in the development of severe bleeding and time to bleeding cessation, a higher occurrence of pulmonary oedema, longer hospitalisation stays [84,85,86]. Considering this platelet transfusions have been advised against by others [84,85,86].

This study has some limitations including small sample sizes and possible selection bias. A larger sample size would allow more confidence in the correlations observed however these observations are in agreement with other published studies [42,48]. The risk of endotoxin contamination was reduced by using depyrogenated glassware, endotoxin free LAL reagent water and tips. Serum tubes were sampled and tested for the presence of endotoxin (<0.080 EU/mL detection limit) (negative controls) and no detectable endotoxin was found. In addition, glucan blocking buffer Glucashield™ was used to prevent non-specific activation of the clotting cascade of LAL reagent by glucans that may have been present. Whole blood contains natural factors which bind endotoxin reducing its detection. The sample size of this study could have been larger to yield more robust data however the study was limited by the maximum number of severe dengue and hospitalised hantavirus cases, matching controls and availability of sufficient volume of patient sera. Future prospective studies on endotoxin should incorporate the monitoring of serum/plasma cytokines to evaluate its co-effect(s) with the immune response. Other limitations and sources of bias do exist for this study including incomplete clinical referral and hantavirus and dengue diagnostic testing which increases the potential to miss mild cases and non-symptomatic dengue and hantavirus cases.

The cytokine production in the presence of endotoxin is key in the pathogenesis of the clinical syndromes of shock and multiple organ failure [40]. DENV NS1 protein functions like LPS in septic shock effecting vascular leakage in the endothelium [87]. The action of elevated serum endotoxin and DENV NS1 levels can possibly exacerbate clinical illness during DENV infection. The role of serum endotoxin and host cytokines on disease severity in other forms of hantavirus clinical presentations other than HPS such as NE and HFRS should be investigated to further understand the host response to hantavirus infections.

This present study represents the first ever study showing an association of serum endotoxin and hantavirus infected patients. In addition, it represents the first evidence of serum endotoxin and its association with dengue severity in the Caribbean. The implications of our studies suggest that viral mediated MT may be one mechanism that enhances both dengue and hantavirus disease severity during acute infection. This also sheds light on other VHF infections such as Ebola virus (EBOV), Marburg virus (MARV), Crimean Congo haemorrhagic fever virus (CCHFV), severe fever with thrombocytopenia syndrome virus (SFTSV), Lassa Fever virus (LFV), and other bunyavirus and filovirus infections and the role serum endotoxin and MT play in disease severity. Further research identifying possible therapeutic targets (e.g., LBP, NS1, CD14) to attenuate disease severity by reducing the effect of MT should be explored.

## Figures and Tables

**Table 1:** Power calculations for the serum endotoxin studies.

	Non-Severe DF	Severe Dengue (SD)	Difference
Mean			0.705 *
Sample size	100	50	
Standard Deviation	2.08	0.6147	
Variance	4.3264	0.377856	

Power = 87.85% by the normal approximation method; * Mean difference = (Group 1 mean serum endotoxin level) − (Group 2 mean serum endotoxin level) from previous study [47].

**Table 2:** Comparison of hospitalised hantavirus patients vs. non-hospitalised hantavirus year, age- and sex-matched patients by sample size, sex, serum endotoxin levels, hantavirus ELISA results and clinical symptoms (2008–2015).

		Hospitalised Hantavirus Patients	Non-Hospitalised Hantavirus Patients	Chi^2^	*p* Value
Number	*n* =	52	104		
Sex	Male	17 (32.7%)	34 (32.7%)		
	Female	35 (67.5%)	70 (67.5%)		
Symptoms	Fever	69.2%	87.5%	10.69 ^Ψ^	<0.01
	Headache	53.8%	74.0%	8.68 ^Ψ^	<0.01
	Gastrointestinal related symptoms	57.7%	53.8%	0.68	>0.01
	Joint pain	34.6%	53.8%	7.31	>0.01
	Myalgia	28.8%	39.4%	2.23	>0.01
	Retroorbital pain	26.9%	29.8%	0.22	>0.01
	Respiratory symptoms	25.0%	6.7%	12.05 ^Ψ^	<0.01
	Rash	11.5%	10.6%	0.05	>0.01
	Thrombocytopenia	11.5%	0%	9.95 ^Ψ^	<0.01
	Liver involvement	9.6%	8.7%	0.06	>0.01
	Bleeding/haemorrhages	5.8%	2.9%	1.05	>0.01
	Hantavirus IgM ELISA positive	52/52 (100%)	104/104 (100%)		
	Hantavirus IgG ELISA positive	2/52 (3.9%)	6/104 (5.8%)		
Endotoxin	Mean (pg/mL)	76.02 (48.10–120.16) ^Ψ^	24.33 (18.07–32.76)		
	Standard Deviation	4.12	3.68		

Ψ—statistically significant (*p* < 0.01). For hospitalised hantavirus patients, respiratory symptoms and thrombocytopenia were all statistically significant clinical symptoms observed when compared with non-hospitalised hantavirus patients. For non-hospitalised patients the frequency of the clinical symptoms fever and headache were statistically significant when compared to hospitalised hantavirus patients.

**Table 3:** The association of serum endotoxin levels and dengue disease severity with comparison of SD, Hospitalised Non-SD and Non-hospitalised Non-SD patients.

		Severe Dengue	Hospitalised Non-Severe Dengue	Non-Hospitalised Non-Severe Dengue
Number		51	51	51
Sex	Male	27 (52.9%)	27 (52.9%)	27 (52.9%)
	Female	24 (47.1%)	24 (47.1%)	24 (47.1%)
Endotoxin	Mean (pg/mL)	86.24 (48.56–153.15)	31.55 (20.63–48.20)	26.96 (17.60–41.30)
	99% CI	(48.56, 153.15) ^†^	(20.62, 48.20) ^†^	(17.60, 41.30)

^†^—statistically significant difference (*p* < 0.01). For SD patients serum mean endotoxin levels was statistically significant higher when compared with hospitalised non-SD patients and non-hospitalised non-SD patients.

**Table 4:** Comparison of serum clinical chemistry parameters for SD and hospitalised DF patients.

		Number of Patients							
Parameter	Normal Range	Severe Dengue (SD)	Hospitalised Non-SD	SD OOR + Non-SD OOR	SD OOR + Non-SD IR	SD IR + Non-SD OOR	SD + Non-SD IR	McNemar’s Test *p* Value	Bonferroni Correction *p* Value
Sodium (Na)	134–144 mmol/L	25	25	5	4	5	11	0.7389	1
Chloride (Cl)	94–104 mmol/L	25	25	11	2	4	8	0.4142	1
Urea	2.5–7.1 mmol/L	15	15	6	1	3	5	0.3173	1
Creatinine	46–87 µmol/L	16	16	4	2	7	3	0.0956	1
T-Bilirubin	3–22 µmol/L	13	13	3	4	3	3	0.7055	1
D-Bilirubin	< 5.1 µmol/L	1	1	0	1	0	0	0.3173	1
ALT	3–35 IU/L	8	8	3	2	3	0	0.6547	1
AST	10–42 U/L	11	11	6	4	1	0	0.1797	1
Albumin	39–49 g/L	4	4	2	0	2	2	0.1573	1
Potassium (K)	2.8–4.1 mmol/L	23	23	3	7	6	6	0.7815	1
Total CO2	22–29 mmol/L	16	16	6	4	3	3	0.7055	1
Anion Gap	4–16	16	16	4	2	6	4	0.1573	1
Uric Acid	2 (2.1)–7 (8.5) mmol/L	0	0	0	0	0	0	N/A	N/A
Alkaline Phosphatase	39–106 IU/L	11	11	1	1	0	9	0.3173	1
Corrected Calcium	2.15–2.51 mmol/L	2	2	0	0	0	2	N/A	N/A
Magnesium (Mg)	0.85–1.1 mmol/L	1	1	0	1	0	0	0.3173	1
Haemoglobin	11.5–16.5 g/dL	20	20	3	5	5	7	1	1
Haematocrit	37–47%	20	20	2	7	6	5	0.7815	1
MCH	27–32 g/dL	20	20	2	7	6	5	1	1
MCV	76–96 g/dL	20	20	0	4	6	10	0.5271	1
RDWCV	11.5–14.5%	20	20	0	6	5	9	0.0253 **	1
BASO%	0–2%	14	14	2	4	3	4	0.7055	1
NEUT%	37–92%	15	15	0	4	6	10	0.3173	1
MONO%	0–12%	18	18	1	3	7	7	0.2059	1
LYM%	10–58.5%	18	18	0	0	5	12	0.3173	1
EOS%	0–7%	18	18	0	0	0	18	N/a	1
WBC	4–11 g/dL	18	18	2	4	3	4	0.5637	1
MCHC	30–35 g/dL	18	18	0	6	2	10	0.1573	1
Red blood cells	3.8–5.8 cells/μL	18	18	2	1	3	8	0.3173	1
Platelets	150–450 × 10^9^/L	17	17	8	5	0	4	0.0253 **	1
Basophils	0.00–0.2 × 10^9^/L	15	15	1	3	7	7	0.3173	1
Neutrophils	2–7.8 × 10^9^/L	15	15	1	5	5	3	1	1
Monocytes	0.0–0.9 × 10^9^/L	18	18	1	3	1	13	1	1
Lymphocytes	0.6–4.1 × 10^9^/L	18	18	1	2	2	13	1	1
Eosinophils	0.0–0.7%	18	18	0	0	0	18	N/A	N/A
IR Range	2–3	3	3	0	0	0	3	N/A	N/A
PTT	23–39 s	4	4	1	3	0	0	0.0833	1
PT	10.9–13.0 s	4	4	3	1	0	0	0.3173	1

** statistically significant *p* < 0.05; OOR—out of range; IR—in range; Partial Thromboplastin time (PTT); millimoles per litre (mmol/L); micromoles per litre (µmol/L); international units per litre (IU/L); grams/litre (g/L).
